# A decade of West Nile virus surveillance in the host and vector populations of Denmark, 2011 to 2023

**DOI:** 10.2807/1560-7917.ES.2025.30.37.2400791

**Published:** 2025-09-18

**Authors:** Ann Sofie Olesen, Charlotta Polacek, Anette Bøtner, René Bødker, Jesper Johannes Madsen, Kasper Thorup, Graham J. Belsham, Thomas Bruun Rasmussen, Louise Lohse

**Affiliations:** 1 Section for Veterinary Virology, Department of Virology & Microbiological Preparedness, Statens Serum Institut, Copenhagen, Denmark; 2 Section for Viral infections and Immunology, Department of Virology & Microbiological Preparedness, Statens Serum Institut, Copenhagen, Denmark; 3 Section for Animal Welfare and Disease Control, Department of Veterinary and Animal Sciences, University of Copenhagen, Frederiksberg, Denmark; 4 Copenhagen Bird Ringing Centre, Natural History Museum of Denmark, University of Copenhagen, Copenhagen, Denmark; 5 Section for Bacteria and Viruses, Department of Veterinary and Animal Sciences, University of Copenhagen, Frederiksberg, Denmark

**Keywords:** Antibodies, *Culex* mosquitoes, flavivirus, poultry, vector-borne, wild birds

## Abstract

**BACKGROUND:**

To spot potential introductions of West Nile virus (WNV) into Denmark, a national surveillance programme for WNV was established in 2011. The relevance of this programme was underscored in the late 2010s, when WNV was detected in areas close to Denmark.

**AIM:**

We describe the Danish WNV surveillance programme and its findings in 2011−2023.

**METHODS:**

The surveillance programme monitors mosquitoes, which are WNV vectors, and some mammalian animals and birds, which are WNV hosts. Surveillance samples are also tested for the closely related Usutu virus (USUV), which, like WNV, is a flavivirus. During the study, WNV and USUV RNAs were sought in 62 bats (sampled in 2022−2023), 5,661 *Culex sp.* mosquitoes (2011−2023), 628 dead wild birds (2011−2014 and 2019−2023), and 492 live birds (2011−2012, 2022−2023). These 492 birds were from among 3,269 live long-distance migratory birds (sampled in 2011−2023) serologically tested for WNV and USUV antibodies. Additionally, 4,978 free-ranging poultry (2011−2023) and 236 horses (2011−2013) were tested serologically.

**RESULTS:**

Neither WNV nor USUV RNA was detected in bats, mosquitoes, or birds, but anti-WNV specific antibodies were detected in migratory birds, one domestic chicken and one imported horse. For migratory birds, competitive ELISAs detected anti-flavivirus antibodies in 3.9% (128/3,269) of tested samples. Across 2011−2023, the annual flavivirus seroprevalence varied from 1−13%. Using virus neutralisation assays on selected samples, anti-WNV or anti-USUV specific antibodies were detected in 25 and 11 bird samples, respectively.

**CONCLUSIONS:**

Findings demonstrate that the concern about virus incursion is well founded and support continued vigilance for WNV.

**Key public health message**

**What did you want to address in this study and why?**

West Nile virus can spread to mammals and birds through bites from *Culex* mosquitos. Human infections by this virus can lead to fever and, occasionally, to neurological symptoms. To be vigilant regarding potential introductions of West Nile virus in Denmark, a national surveillance programme for this virus was established in 2011. Later, detections of West Nile virus in European countries close to Denmark highlighted the relevance of this programme and its findings, which we present.

**What have we learnt from this study?**

The Danish West Nile surveillance programme focuses on mosquitoes, which are vectors of the virus, and some animal species, which can host it. We describe the programme and its results from 2011 to 2023. During this time, West Nile virus was not detected in dead wild birds, bats or *Culex* mosquitoes sampled in Denmark, but antibodies signalling past infection with the virus (or vaccination) were found in migratory birds, an outdoor domestic chicken, and one imported horse.

**What are the implications of your findings for public health?**

To our knowledge, this is the first study of West Nile virus in animal populations in Denmark. The surveillance programme did not detect West Nile virus in Denmark from 2011 to 2023 but the study results indicate that the risk of incursion exists. Surveillance of West Nile virus within insects that can transmit the virus and some bird or mammalian species, can allow early warning and timely implementation of prevention measures to protect humans and other animals.

## Introduction

West Nile virus (WNV), like Usutu virus (USUV), is a vector-borne enveloped positive-sense, single stranded RNA virus in the Japanese encephalitis virus (JEV) serocomplex within the family *Flaviviridae* [1,2]. Transmission of WNV, as USUV, mostly occurs through bites of infected mosquitoes − mainly of the *Culex* genus, which serve as vectors for the virus. Birds can become infected by both USUV and WNV and are considered reservoir hosts; humans and other mammals, such as equids, are considered dead-end hosts [2].

People infected with WNV can be asymptomatic, however, this virus can also cause a febrile illness termed West Nile fever (WNF); moreover, in some instances, more severe infections manifest as neuroinvasive disease (WNND) [2,3]. Usutu virus causes mortality in certain wild bird species [2,4] but in humans, only few cases of symptomatic USUV infections have been reported; these occurred in immunocompromised and/or older adults and presentation included febrile illness or neurological symptoms [2,4].

At the European Union (EU) level, infections by WNV have been notifiable since 2008 [5]; USUV infections are not uniformly notifiable across the EU [4,5].

Currently, WNV is classified into as many as nine genetic lineages [6]. Lineages 1 and 2 are the most widespread globally and are mainly responsible for human cases of WNF and WNND [3]. In Europe, strains of lineage 1 were isolated from patients and mosquitoes as early as the 1960s. Such strains subsequently caused sporadic infections and intermittent outbreaks in animals and humans [7]. Lineage 2 was first detected in Europe in 2004, in Hungary. Thereafter, viruses of this lineage spread in Central Europe in 2008/09 [8,9], subsequently causing major outbreaks in other parts of the continent [9], such as in Greece in 2010 [10].

In 2011, the European Centre for Disease Control and Prevention (ECDC) started weekly surveillance updates on WNV between June and November on a yearly basis [11]. The same year, while there was no evidence of WNV autochthonous human infections in Denmark [12], a Danish national WNV surveillance programme focusing on animals was established in the country to increase vigilance regarding potential introductions of this virus. This Danish programme monitors WNV in non-human hosts and in *Culex* vector populations. Samples submitted to the WNV programme in Denmark are also tested for USUV, which has been detected in birds in neighbouring Germany since 2011 [13], and which shares the same bird-mosquito life cycle, often co-existing with WNV geographically [14].

While, according to a report from 2021 [9], northern Europe has not been regarded as endemic for WNV, circulation of WNV in the late 2010s has been detected in areas increasingly close to Denmark. In Germany, which borders the south of Denmark, WNV lineage 2 was identified in both *Culex pipiens* mosquitoes in 2019 [15], and in birds in 2018, 2019 and 2020 [8,16]. In 2024, infections in birds and horses were also reported in the northwestern part of Germany, less than 50 km from the Danish border [17]. These events underline the relevance of the Danish WNV surveillance and of its findings.

Here we describe the national surveillance for WNV in Denmark during the 2011 to 2023 period and its results regarding WNV and USUV in vector and animal host populations.

## Methods

### Samples submitted to the surveillance programme

Samples that were submitted to the Danish programme from 2011 to 2023 included *Culex sp.* mosquitoes, as well as samples from free-ranging domestic birds (outdoor poultry) and live, wild, long-distance migratory birds. Samples from wild birds found dead were also provided in 2011−2014 and 2019−2023. Moreover, serum samples from horses, which can also be infected with WNV [18], were included in the early years of the programme (2011−2013). From 2022 onwards, brain tissue samples collected from bats in Denmark were included in the WNV and USUV analyses since USUV had been detected in *Pipistrellus* bats in 2013 in an area endemic for the virus in Germany (southwest Germany, Rhineland-Palatinate federal state) [19]. As further described, some submitted samples were analysed for the presence of USUV and WNV genetic material (virus testing), some for presence of antibodies (serological testing), and some for both.

#### Samples for virus testing

**Bats**: In 2022 and 2023, brain tissue samples from 20 and 42 bats respectively, collected under the passive surveillance programme for rabies [20,21], were submitted to the WNV surveillance programme. While most samples were from *Pipistrellus* bats, some originated from other bat species. As all bat samples submitted via the passive surveillance programme for rabies to the WNV programme are analysed, the species and numbers of bats tested during the 2 years included 35 soprano pipistrelle bats (*Pipistrellus pygmaeus*), six brown long-eared bats (*Plecotus auritus*), five parti-coloured bats (*Vespertilio murinus*), three serotine bats (*Eptesicus serotinus*)*,* one Brandt's bat (*Myotis brandtii*), one pond bat (*Myotis dasycneme*) and 11 unidentified bats (species could not be determined). The locations (at the municipality level) from which the bats originated are shown in the Figure (panel A).

**Figure:**
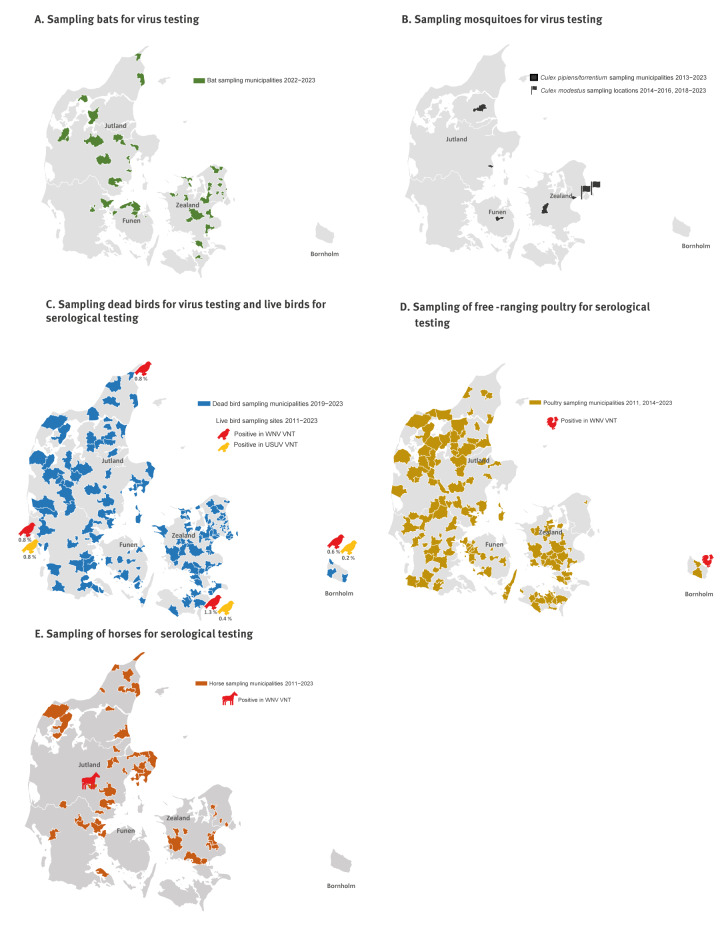
Maps indicating locations of each species from which the different samples for the West Nile virus surveillance programme originated, Denmark, 2011−2023

Created using Excel (Microsoft).

USUV: Usutu virus; VNT: viral neutralisation test; WNV: West Nile virus.

Panel A. Sampling locations from which bats were submitted to the Danish WNV surveillance programme during 2022−2023; the locations are depicted at municipality level (indicated in green).

Panel B. Five municipalities (indicated in black) where *Culex pipiens/torrentium* mosquitoes were sampled in five respective private gardens, during 2013−2023. Locations for *Cx. Modestus* sampling (2014−2016, 2018−2023) are indicated by flags.

Panel C. Municipalities (in blue) where dead wild birds were sampled during 2019−2023 (locations of dead wild birds sampling in 2011−2014 are not available), as well as the four locations used for ringing and sampling live migratory birds during 2011−2023. The four bird ringing locations are shown as bird icons, with red indicating detection of anti-WNV antibodies (by WNV VNT) and yellow indicating detection of anti-USUV antibodies (by USUV VNT); the percentages of serum samples that scored positive in the WNV and USUV VNTs at each location are shown.

Panel D. Municipalities (indicated in gold) where poultry was sampled, during 2011 and 2014−2023 (sampling locations for poultry from 2012 and 2013 are not available). The red-coloured bird indicates the location of a single chicken found seropositive for WNV (by WNV VNT), which was detected in 2020 on the island of Bornholm.

Panel E. Municipalities from where horse samples were submitted, 2011−2013. The red-coloured horse indicates the location of a single imported horse found seropositive for WNV (by WNV VNT), which was identified in 2011.

**Mosquitoes**: *Culex* mosquitoes collected as part of the Danish vector surveillance programme [22], were provided to the WNV programme. In total, during 2011−2023, regardless of species, 5,661 *Culex* mosquitoes were obtained. *Culex pipiens* and *Cx. torrentium* were trapped using CO_2_, octenol and warm air baited Mosquito Magnet traps that collected mosquitoes for ca 72 hours; the traps were operated on a weekly basis during the period from late spring to early autumn. During 2011−2012, traps were positioned randomly in gardens, parks and on farms with production animals [23], but from 2013 and onwards, in predominantly the same five, randomly selected, private gardens in Denmark (Figure, panel B). *Culex modestus* were collected (from 2014−2016 and 2018−2023) either using Mosquito Magnet traps or by human landing catch (i.e. human volunteers catch mosquitoes that land on them) near known *Cx. modestus* hotspots along the shore of the Køge Bay area south of Copenhagen [24].

**Dead wild birds**: From the wild bird population, brain tissue samples from dead wild birds submitted to the passive surveillance programme for avian influenza virus (AIV) [25-27] were included in the WNV surveillance programme from 2011−2014 and again from 2019−2023. In total 628 samples (one per bird) were collected during the study. The primary focus was birds known to present clinical manifestations of WNV or USUV infections, i.e. birds of prey and passerines. The locations (at the municipality level) from which the dead wild birds originated from 2019 to 2023 are shown in the Figure (panel C) − sample locations for dead wild birds from 2011−2014 are not available.

#### Samples for virus testing and/or serology

**Live wild birds**: The wild bird population was primarily monitored within live migratory birds. Samples were taken in spring during 2011−2023, at migratory resting hotspots and 99% of these (3,238/3,269) were from bird species overwintering in sub-Saharan Africa [28]. In total 3,269 live migratory birds were sampled. The birds were sampled at four different geographical locations in Denmark (Figure, panel C). The migratory birds were caught using mist nets in connection with the national bird ringing programme [29] and small volumes of blood were drawn for laboratory analysis before releasing the birds into the wild again.

**Free-ranging (outdoor) poultry**: From free-ranging (outdoor) poultry, serum samples were collected annually under the active surveillance programme for AIV and included in the Danish WNV surveillance programme (from 2011−2021) [25]. Due to implementation of the new Animal Health Law within the European Union (EU) [30], submission of these samples was discontinued in 2021. Hence, from 2022, samples from free-ranging domestic chickens could only be obtained by voluntary submissions directly to the WNV surveillance programme. The locations (at a municipality level) from which the poultry originated are shown in the Figure (panel D) − sample locations for poultry samples from 2012−2013 are not available. Overall, during 2011−2023, the Danish WNV surveillance programme obtained 4,978 poultry samples.

**Horses**: In 2011, 2012 and 2013, serum samples from 136, 81 and 19 healthy horses, respectively were collected. In total, 236 were submitted to the WNV surveillance programme on a voluntary basis [31]. In 2011, 69 serum samples were collected from horses in the Copenhagen area (from the Guard Hussar Regiment), and 67 serum samples were submitted from various veterinary hospitals in Denmark during August and September. In 2012 and 2013, samples were submitted from veterinary hospitals only. The locations (at a municipality level) from which the horses originated, if disclosed, are shown in the Figure (panel E).

### Laboratory testing of submitted samples

#### Testing for WNV and USUV by reverse-transcription quantitative PCR

For the reverse-transcription quantitative (RT-q)PCR, tissue homogenates were prepared from all samples. Brains from dead wild birds and bats were stored at − 80 °C and then homogenised in Minimum Essential Medium (MEM) (Sigma-Aldrich) (20% weight/volume) by vigorous vortexing. Trap-collected mosquitoes were classified to species level by stereomicroscopy at room temperature, and the identified *Culex* mosquitoes were then kept at − 20 °C until laboratory analyses. *Culex modestus*, collected by human landing catch, were kept at − 20 °C following collection. Mosquitoes were pooled (≤ 25 per pool) according to location, month and species. To each mosquito pool, MEM (0.5 mL) or RNAse-free water was added and homogenisation was performed using a 3 mm stainless steel bead (Dejay Distribution) in a TissueLyser II (Qiagen) for 1 minute at 25 Hz.

Brain and mosquito homogenates were centrifuged, and supernatants were used for RNA extraction. Blood pellets obtained from selected blood samples from migratory birds were resuspended in 250 µL phosphate buffered saline (Sigma-Aldrich) prior to extraction of RNA. Viral RNA was extracted using the GeneJet RNA Purification Kit (Thermo Fischer Scientific) or the MagNA Pure 96 system (Roche), the latter with the DNA/Viral NA S.V. 2.0 and Viral NA Plasma extern lysis S.V. 3.1 protocol.

Slightly modified versions of the assays described by Linke et al. (for WNV) [32] and Cavrini et al. (for USUV) [33] were used for RT-qPCR. As an internal control for amplification of nucleic acids, an assay targeting beta-actin mRNA described by Toussaint et al. (2007) [34] was used for all samples. The assay was developed to target ruminant genomic material [34], but it also yields positive results for the tested bat-, mosquito- and bird samples. Reverse transcription and amplification were performed on the Mx3005P qPCR system (Agilent Technologies) or the CFX Opus Real-Time PCR system (Bio-Rad).

#### Serology

Serum samples from migratory birds, free-ranging poultry, and horses were tested for the presence of antibodies against WNV using a commercial immunoglobulin (Ig)G ELISA; the ID-Screen West Nile Competition Multi-species (Innovative Diagnostics; IDvet), allowing detection of antibodies from various species. However, the IgG-ELISA was recently re-commercialised by the manufacturer under the name ID Screen Flavivirus Competition due to its cross-reactivity to other flaviviruses in the JEV serocomplex. The assay was carried out according to the manufacturer´s instructions. All serum samples were stored at − 20 °C prior to analysis.

Since the ID-Screen West Nile Competition Multi-species (IDvet) cross-reacts with antibodies against other flaviviruses of the JEV serocomplex, confirmatory follow-up was needed for positive and borderline samples. Hence, if sufficient serum was left for further characterisation, samples from migratory birds and poultry were sent to the European Reference Laboratory for Equine Diseases (EURL-ANSES, France) for flavivirus discrimination from 2013 until 2023. Samples from migratory birds were tested using the WNV/USUV virus neutralisation test (VNT) using the methods described in chapter 3.1.25 of the manual of Diagnostic Tests and Vaccines for Terrestrial Animals, which is published by the World Organisation for Animal Health [35]. Poultry samples were tested using the WNV VNT only, at the reference laboratory. From 2011 and 2012 only, the WNV VNT was performed on ELISA positive serum samples (migratory birds and horses). During those 2 years the VNT was carried out at the former National Veterinary Institute (Technical University of Denmark) using the Israeli WNV IS-98-ST1 strain on Vero NK cells for specific WNV antibody detection (both kindly provided by Sylvie Lecollinet at EURL-ANSES, France).

## Results

### Bats

WNV and USUV RNA was not detected in brain material from the 62 bats that were analysed in 2022 and 2023.

### *Culex* mosquitoes

WNV and USUV RNA was not detected in the sampled Danish vector population (5,661 mosquitoes, Table1) sampled from 2011 until 2023.

**Table 1:** Overview of collected mosquito species, which were tested by RT-qPCR, Denmark, 2011−2023 (n = 5,661 samples)

Mosquito species	Numbers of samples														
	2011	2012	2013	2014	2015	2016	2017	2018	2019	2020	2021	2022	2023	Total	Testing positives in RT-qPCR
*Culex pipiens/torrentium*	884	306	116	190	250	489	275	24	206	216	3	43	380	3,382	0
*Cx. modestus*	0	0	0	1,291	1	41	0	12	125	24	409	372	4	2,279	0
**Total**	**884**	**306**	**116**	**1,481**	**251**	**530**	**275**	**36**	**331**	**240**	**412**	**415**	**384**	**5,661**	**0**

RT-qPCR: reverse-transcription quantitative real-time polymerase chain reaction.

### Dead wild birds

WNV and USUV RNAs were not detected in brain material from the 628 dead wild birds submitted to the surveillance programme. Submissions of birds varied from 11 up to 150 birds per year (Table 2).

**Table 2:** Species origin of brain samples from dead wild birds, tested by RT-qPCR, Denmark, 2011−2014, 2019−2023 (n = 628 samples)

Order	Family (number of species sampled)	Number of samples										
		2011	2012	2013	2014	2019	2020	2021	2022	2023	Total	Positive in RT-qPCR
Accipitriformes	Accipitridae (7)	NA	0	4	13	35	55	61	28	23	219	0
	Pandionidae (1)	NA	0	0	0	0	0	1	0	0	1	0
Anseriformes	Anatidae (4)	NA	2	5	5	0	0	0	0	0	12	0
Charadriiformes	Charadriidae (1)	NA	0	7	0	0	9	0	0	0	16	0
	Laridae (3)	NA	0	1	6	2	0	2	0	0	11	0
	Scolopacidae (1)	NA	0	1	0	0	0	0	0	0	1	0
	Columbidae (2)	NA	0	2	0	0	0	0	1	0	3	0
Falconiformes	Falconidae (3)	NA	0	13	1	4	24	17	3	7	69	0
	Accipitridae (1)	NA	0	0	0	2	2	3	2	2	11	0
Galliformes	Phasianidae (1)	NA	0	0	1	0	0	0	0	0	1	0
Passeriformes	Alaudidae (1)	NA	0	0	0	0	0	1	0	0	1	0
	Corvidae (8)	NA	0	1	4	7	18	41	17	6	94	0
	Fringillidae (4)	NA	2	0	2	14	1	0	0	0	19	0
	Paridae (1)	NA	0	0	0	1	0	0	0	1	2	0
	Passeridae (1)	NA	0	0	2	0	0	2	0	0	4	0
	Prunellidae (1)	NA	0	0	0	1	0	0	0	0	1	0
	Regulidae (1)	NA	0	0	0	1	0	0	0	0	1	0
	Sturnidae (1)	NA	0	5	0	0	0	0	0	0	5	0
	Turdidae (3)	NA	0	0	2	17	18	7	0	1	45	0
Strigiformes	Strigidae (3)	NA	0	0	2	0	3	6	4	4	19	0
	Sylviidae (1)	NA	0	0	0	1	0	0	0	0	1	0
	Tytonidae (1)	NA	0	0	0	2	1	1	0	0	4	0
Suliformes	Phalacrocoracidae (1)	NA	0	0	0	0	0	0	1	0	1	0
Unidentified^a^	NA	69	7	0	1	2	0	8	0	0	87	0
**Total**		**69**	**11**	**39**	**39**	**89**	**131**	**150**	**56**	**44**	**628**	**0**

NA: not applicable; RT-qPCR: reverse transcription quantitative real-time polymerase chain reaction.

^a^ Recorded variously (e.g. as crow, owl, goose, swallow in 2011), but specific numbers for each species were not specified in 2011.

### Live migratory birds

From 2011 to 2023, serum samples were collected from 79−333 migratory birds per year, totalling 3,269 birds (Table 3). From each ringing site, 368 samples (Northern Denmark), 459 samples (Southern Denmark), 746 samples (Western Denmark) and 1,696 samples (Eastern Denmark) were submitted (Figure, panel C). Samples were primarily obtained from migratory birds within the Order Passeriformes (3,252/3,269; 99.5%) (Table 3).

**Table 3:** Overview of serum samples from migratory birds collected and tested by competitive ELISA, Denmark, 2011−2023 (n = 3,269 samples)

Order	Family (number of species sampled)	Number of samples														
		2011^a^	2012^a^	2013	2014	2015	2016	2017	2018	2019	2020	2021	2022^a^	2023^a^	Total	Positive in flavivirus ELISA
Cuculiformes	Cuculidae (1)	0	0	0	0	0	0	0	0	0	0	1	0	0	1	0
Passeriformes	Acrocephalidae (4)	13	24	12	3	22	4	46	1	7	8	12	22	32	206	3
	Fringillidae (1)	0	11	1	0	0	0	0	0	0	0	0	1	0	13	0
	Hirundinidae (3)	2	1	0	0	7	0	0	0	2	0	0	2	0	14	1
	Laniidae (1)	1	5	11	8	22	3	5	2	2	5	2	0	0	66	10
	Locustellidae (1)	0	0	0	0	0	1	0	0	0	0	1	1	0	3	0
	Motacillidae (1)	1	1	6	14	10	4	3	5	10	5	11	7	5	82	5
	Muscicapidae (10)	29	74	84	100	70	124	19	122	84	114	110	88	33	1,051	23
	Oriolidae (1)	1	0	0	0	0	0	0	0	0	0	0	0	0	1	0
	Phylloscopidae (2)	0	2	5	13	8	7	1	2	3	1	1	0	1	44	2
	Sylviidae (5)	31	82	80	149	141	145	176	184	204	131	194	112	143	1,772	83
Piciformes	Turdidae (2)	1	1	0	0	0	0	0	0	0	0	0	0	0	2	0
Piciformes	Picidae (1)	0	0	2	0	0	0	0	0	1	4	1	3	2	13	1
Unidentified		0	0	0	0	0	0	0	0	1	0	0	0	0	1	0
**Total**		**79**	**201**	**201**	**287**	**280**	**288**	**250**	**316**	**314**	**268**	**333**	**236**	**216**	**3,269**	**128**

ELISA: enzyme-linked immunosorbent assay.

^a^ In 2011, 2012, 2022 and 2023 either all or selected samples (79, 201, 120 and 92, respectively) were additionally analysed for viral RNA on blood pellets.

Using pathogen-specific RT-qPCRs, WNV and USUV RNAs were not detected in blood pellets from migratory birds (79 samples in 2011, 201 samples in 2012, 120 samples in 2022, and 92 samples in 2023).

Using competitive ELISA, collectively, among the 3,269 sampled migratory birds from 2011 to 2023, 128 (3.92%; binomial confidence intervals: 3.25−4.58%) tested positive for antibodies against flaviviruses (Table 3 and Table 4), while 35 samples were inconclusive (Table 4). From these positive or inconclusive samples, 70 samples were further tested in confirmatory WNV VNT and 45 samples in confirmatory USUV VNT.

**Table 4:** West Nile virus and Usutu virus serology in migratory birds determined by competitive ELISA and VNTs, Denmark, 2011−2023 (n = 3,269 samples)

Year		Number of samples				Seroprevalence % (flavivirus ELISA)
		Analysed	Positive	Inconclusive^a^	Negative	
2011	Competitive ELISA	79	3	2	74	4
	WNV VNT	5	3	0	2	NA
	USUV VNT	0	ND	ND	ND	NA
2012	Competitive ELISA	201	4	0	197	2
	WNV VNT	4	4	0	0	NA
	USUV VNT	0	ND	ND	ND	NA
2013	Competitive ELISA	201	9	0	192	4
	WNV VNT	2	1	0	1	NA
	USUV VNT	0	ND	ND	ND	NA
2014	Competitive ELISA	287	3	3	281	1
	WNV VNT	0	ND	ND	ND	NA
	USUV VNT	0	ND	ND	ND	NA
2015	Competitive ELISA	280	11	1	268	4
	WNV VNT	0	ND	ND	ND	NA
	USUV VNT	0	ND	ND	ND	NA
2016	Competitive ELISA	288	6	0	282	2
	WNV VNT	5	4^b^	1	0	NA
	USUV VNT	5	2^b^	1	2	NA
2017	Competitive ELISA	250	14	2	234	6
	WNV VNT	10	7^b^	1	2	NA
	USUV VNT	7	1^b^	2	4	NA
2018	Competitive ELISA	316	8	2	306	3
	WNV VNT	6	2^b^	2	2	NA
	USUV VNT	6	2^b^	1	3	NA
2019	Competitive ELISA	314	13	2	299	4
	WNV VNT	3	1	1	1	NA
	USUV VNT	3	0	0	3	NA
2020	Competitive ELISA	268	7	1	260	3
	WNV VNT	4	1	0	3	NA
	USUV VNT	0	ND	ND	ND	NA
2021	Competitive ELISA	333	14	5	314	4
	WNV VNT	7	1	5	1	NA
	USUV VNT	0	ND	ND	ND	NA
2022	Competitive ELISA	236	31	14	191	13
	WNV VNT	19	0	0	19	NA
	USUV VNT	19	2	0	17	NA
2023	Competitive ELISA	216	5	3	208	2
	WNV VNT	5	1^b^	4	0	NA
	USUV VNT	5	4^b^	0	1	NA
**Total**	**Competitive ELISA**	**3,269**	**128**	**35**	**3,106**	**4**
	**WNV VNT**	**70**	**25**	**14**	**31**	**NA**
	**USUV VNT**	**45**	**11**	**4**	**30**	**NA**

ELISA: enzyme-linked immunosorbent assay; NA: not applicable; ND: analysis not done, most often due to insufficient sample material; USUV: Usutu virus; VNT: virus neutralisation test; WNV: West Nile virus.

^a^ ‘Inconclusive’ refers to borderline samples (competitive-ELISA and VNT).

^b^ Some of these samples were positive for antibodies against both WNV and USUV using VNT.

Results from competitive-ELISA for all tested serum samples from migratory birds, and WNV and USUV VNT results.

On a yearly basis (except in 2022), 1−6% of the sampled migratory birds tested positive for antibodies against flaviviruses in the competitive ELISA (Table 4).

Of the 70 and 45 ELISA-positive samples tested by VNT for antibodies to WNV and USUV respectively, 25 of 70 (36%) were confirmed to be WNV antibody positive and 11 of 45 (24%) USUV antibody positive. The 25 WNV VNT positive samples, constituted 0.8% of the total 3,269 wild bird samples analysed by competitive ELISA, while the 11 USUV VNT positive samples were 0.3% of this total.

The 25 samples in which WNV specific antibodies were detected, were obtained from birds within 10 species of the order Passeriformes, namely five common whitethroats (*Curruca communis*), six garden warblers (*Sylvia borin*), four common redstarts (*Phoenicurus phoenicurus*), one willow warbler (*Phylloscopus trochilus*)*,* two lesser whitethroats (*Curruca curruca*), two red-backed shrikes (*Lanius collurio*), one tree pipit (*Anthus trivialis*), one spotted flycatcher (*Muscicapa striata*), two blackcaps (*Sylvia atricapilla*), and one wood warbler (*Phylloscopus sibilatrix*). The 11 samples in which USUV specific antibodies were detected originated from five passerine bird species, namely four garden warblers (*Sylvia borin*) (of these 3 were also positive for WNV antibodies), three common whitethroats (*Curruca communis*; of these one was also positive for WNV antibodies), two icterine warblers (*Hippolais icterina*), one wood warbler (*Phylloscopus sibilatrix*; also positive for WNV antibodies) and one northern wheatear (*Oenanthe oenanthe*). Samples defined as cross-reacting here (i.e. the three *Sylvia borin*, the one *Curruca communis* and the one *Phylloscopus sibilatrix*) had less than a fourfold neutralising titre difference in the WNV VNT and USUV VNT. Neutralisation titres in the five birds reported as positive in both assays ranged from 1:80 to 1:160 (WNV VNT) and 1:10 to 1:80 (USUV VNT). Samples in which antibodies specific for WNV were detected originated from all four ringing sites, while specific antibodies for USUV were detected in samples from three ringing sites (Figure, panel C). The majority of the WNV and USUV-positive birds are long-distance migrants, overwintering in sub-Saharan Africa. Indeed, nine of 10 species in which antibodies specific for WNV were detected, and all five species in which antibodies specific for USUV were detected migrate to Sub-Saharan Africa. Only *Sylvia atricapilla* can winter further north, in the Mediterranean region.

In the year 2022 alone, 31 serum samples (13%) obtained from migratory birds tested positive for antibodies against flaviviruses in the competitive ELISA (Table 4). This higher incidence coincided with a significant increase in the human WNV cases acquired in Europe in 2022 [26]. The elevated incidence did not, however, seem to reflect an increase in anti-WNV or anti-USUV specific antibodies in birds sampled that year, as zero (0%) and two (10.5%) of 19 ELISA-positive samples tested positive in discriminatory WNV and USUV VNT, respectively (Table 4). Fluctuations in the proportion of birds testing positive using the competitive ELISA were also observed prior to 2022 (from 2 to 6% depending on the year), however this could reflect the total number of samples from each bird species per year as shown in the Supplementary Material. When calculating the proportion of samples per species that tested positive per year, there appeared to be an increase in the proportion of positive birds for five species in 2022 compared to previous years (Supplementary Material, Table 5). These five species included the common whitethroat (*Curruca communis*), the garden warbler (*Sylvia borin*), the lesser whitethroat (*Curruca curruca*), the icterine warbler (*Hippolais icterina*) and the European pied flycatcher (*Ficedula hypoleuca*) (Table 5).

**Table 5:** Overview of five relevant species^a^ of migratory birds in which antibodies against flaviviruses were detected using competitive ELISA, with findings stratified by year, Denmark, 2011–2023 (n = 1,198 samples)

Year	*Curruca communis* **common whitethroat**			*Sylvia borin* **garden warbler**			*Curruca curruca* **lesser whitethroat**			*Hippolais icterina* **icterine warbler**			*Ficedula hypoleuca* **European pied flycatcher**		
	Number of samples		Proportion of ELISA-positive samples (95%CI)	Number of samples		Proportion of ELISA-positive samples (95%CI)	Number of samples		Proportion of ELISA-positive samples (95%CI)	Number of samples		Proportion of ELISA-positive samples (95%CI)	Number of samples		Proportion of ELISA-positive samples (95%CI)
	ELISA tested	Positive		ELISA tested	Positive		ELISA tested	Positive		ELISA tested	Positive		ELISA tested	Positive	
2011	14	0	0 (0–0.22)	9	1	0.11 (0.02–0.43)	2	0	0 (0–0.66)	11	0	0 (0–0.26)	7	0	0 (0–0.35)
2012	8	1	0.12 (0.02–0.47)	31	1	0.03 (0.01–0.16)	7	0	0 (0–0.35)	20	0	0 (0–0.16)	3	0	0 (0–0.56)
2013	25	5	0.20 (0.09–0.39)	34	2	0.06 (0.02–0.19)	2	0	0 (0–0.66)	8	0	0 (0–0.32)	7	0	0 (0–0.35)
2014	16	0	0 (0–0.19)	44	2	0.05 (0.01–0.15)	5	0	0 (0–0.43)	2	0	0 (0–0.66)	17	0	0 (0–0.18)
2015	27	2	0.07 (0.02–0.23)	84	2	0.02 (0.01–0.08)	15	0	0 (0–0.20)	13	0	0 (0–0.23)	4	0	0 (0–0.49)
2016	10	1	0.10 (0.02–0.40)	30	3	0.10 (0.03–0.26)	14	0	0 (0–0.22)	3	0	0 (0–0.56)	25	0	0 (0–0.13)
2017	40	7	0.17 (0.09–0.32)	30	2	0.07 (0.02–0.21)	32	2	0.06 (0.02–0.20)	22	0	0 (0–0.15)	3	0	0 (0–0.56)
2018	20	2	0.10 (0.03–0.30)	52	1	0.02 (0–0.10)	23	1	0.04 (0.01–0.21)	0	0	NA	6	0	0 (0–0.39)
2019	16	2	0.12 (0.03–0.36)	35	0	0 (0–0.10)	43	2	0.05 (0.01–0.15)	4	0	0 (0–0.49)	2	0	0 (0– 0.66)
2020	6	0	0 (0–0.39)	33	1	0.03 (0.01–0.15)	25	0	0 (0–0.13)	6	0	0 (0–0.39)	1	0	0 (0–0.79)
2021	34	3	0.09 (0.03–0.23)	28	2	0.07 (0.02–0.23)	62	1	0.02 (0–0.09)	8	0	0 (0–0.32)	15	0	0 (0–0.20)
2022	22	7	0.32 (0.16–0.53)	11	3	0.27 (0.10–0.57)	15	6	0.40 (0.20–0.64)	16	3	0.19 (0.07–0.43)	8	1	0.12 (0.02–0.47)
2023	24	3	0.12 (0.04–0.31)	21	0	0 (0–0.15)	19	0	0 (0–0.17)	17	0	0 (0–0.18)	2	0	0 (0–0.66)
**Total**	**262**	**33**	**0.13** (**0.09–0.17)**	**442**	**20**	**0.05** (**0.03–0.07**)	**264**	**12**	**0.05** (**0.03–0.08**)	**130**	**3**	**0.02** (**0.01–0.07**)	**100**	**1**	**0.01** (**0–0.05**)

CI: confidence intervals; ELISA: enzyme-linked immunosorbent assay; NA: not applicable.

^a^ The overview of all species of migratory birds in the study, in which antibodies against flaviviruses were detected using competitive ELISA is described in the Supplementary Material.

### Free-ranging poultry

Approximately 400 serum samples from free-ranging poultry were collected annually under the active surveillance programme for AIV and included in the Danish WNV surveillance programme (from 2011 to 2021). After discontinuation of the AIV programme in 2021, submissions to the WNV surveillance programme (now on a voluntary basis) decreased drastically during 2022 and 2023 (Table 6).

**Table 6:** Species origin of serum samples from free-ranging poultry, tested by competitive ELISA, Denmark, 2011−2023 (n = 4,978 samples)

Order	Family	Genus and species	Common name	Number of samples													Total number of	
				2011	2012	2013	2014	2015	2016	2017	2018	2019	2020	2021	2022	2023	Samples	Samples positives in flavivirus ELISA
Galliformes	Phasianidae	*Phasianus colchicus*	Pheasants	310	249	226	154	140	188	190	190	160	206	175	0	0	2,188	0
		*Gallus gallus domesticus*	Chickens	21	0	0	1	10	20	60	130	70	139	250	91	79	871	2
		*Perdix perdix*	Partridges	10	13	20	4	33	22	10	10	10	39	5	0	0	176	0
		*Meleagris gallopavo domesticus*	Turkeys	0	0	10	0	10	30	10	0	0	0	0	0	0	60	0
Anseriformes	Anatidae	*Anas platyrhynchos*	Mallard ducks	20	160	120	220	182	97	140	161	140	80	130	0	0	1,450	0
		*Anas* (no species identified)	Ducks	40	20	19	0	0	0	0	0	20	0	0	0	0	99	0
		*Anser* spp.	Geese	20	0	0	20	20	20	0	0	0	0	0	0	0	80	0
Charadriiformes	Scolopacidae	*Tringa totanus*	Common redshank	0	0	0	1	0	19	0	0	0	0	0	0	0	20	0
Unidentified^a^	NA	NA	NA	20	14	0	0	0	0	0	0	0	0	0	0	0	34	0
**Total**				**441**	**456**	**395**	**400**	**395**	**396**	**410**	**491**	**400**	**464**	**560**	**91**	**79**	**4,978**	**2**

ELISA: enzyme-linked immunosorbent assay, NA: Not applicable.

^a^ Birds from 2011 and 2012 were recorded as partridge/pheasant but were not specified further.

In just two of 4,978 serum samples collected from outdoor poultry from 2011 until 2023, antibodies against flaviviruses were detected by competitive ELISA (Table 6). The two samples in which antibodies against flaviviruses were detected were obtained in 2020 and 2021. Both samples originated from free-ranging chickens, from which 139 and 250 serum samples were analysed in 2020 and 2021, respectively (Table 6). The sample from 2020 also tested positive for anti-WNV specific antibodies in WNV VNT at the EURL for Equine diseases (ANSES, France) with a titre of 160. This sample originated from the Island of Bornholm (Eastern Denmark, Figure, panel D). The sample from 2021 tested negative in the confirmatory WNV VNT analysis performed at the EURL.

### Horses

During 2011−2013, 236 sera from healthy domestic horses were included in the WNV surveillance programme. In 2011, a single horse tested positive for antibodies against WNV using competitive ELISA and then confirmatory WNV VNT (Figure, panel E). The animal was imported from the United States in 2009 and had an undisclosed WNV vaccination and infection history [31,36].

## Discussion

In this study, WNV RNA was not detected in wild birds, bats or *Culex* mosquitoes in the reporting period (from 2011 to 2023). However, antibodies against the virus were detected in migratory birds, a domestic free-ranging bird (outdoor poultry) and in one imported horse. Surveillance for WNV in Denmark is becoming increasingly relevant, especially in the light of developments in the 2024 WNV transmission season, with symptomatic cases in animals less than 50 km south of the Danish−German border [17] and the first detections of WNV in Latvia and Estonia [37].

In the reporting period from 2011−2023, a seroprevalence for anti-flavivirus antibodies of 1−13% was detected in migratory birds, using the competitive ELISA. The seroprevalences reported from Denmark correspond with reported seroprevalences for anti-WNV antibodies from Germany and Sweden. The study from Germany reported a flavivirus seroprevalence of approximately 7% in wild birds sampled in 2019 and 2020 from (at that time) non-endemic regions of the country, close to Denmark. Follow-up samples from 2020 showed that ca. 0.3% (one bird of 365) tested positive in the WNV VNT [16], which is also in line with the results obtained from Denmark in 2020. It should be noted, that the bird that tested positive in WNV VNT from Germany in 2020 was not a long-distance migratory bird (a long-eared owl, *Asio otus*) [16]. In a study where 1,935 migratory birds were sampled in 2005 and 2006 in southeastern Sweden, seroprevalences of 2.4% and 0.1% were detected by ELISA and the WNV VNT, respectively, in these birds [38]. In a later study from Sweden, seroprevalences of only 0.51% (ELISA) and 0.41% (WNV VNT) were reported from 1,175 migratory birds sampled in 2021 [39], which, for the flavivirus seroprevalences determined by ELISA, is lower when compared to our study, where we detected a seroprevalence of 4% in 2021 using the ELISA. The difference observed could be due to several factors, e.g. year-to-year variation or different ringing sites or different laboratory methods. Interestingly, we used the same commercial ELISA as reported from the Swedish study [39].

We observed a higher incidence of ELISA positive birds in 2022 compared to other years within the reporting period. This higher incidence coincided with a considerable increase in the human WNV cases observed in Europe in 2022 [40]. However, the lack of neutralising antibodies in most samples (19 for WNT VNT and 17 for USUV VNT) among the 19 ELISA positive samples collected in 2022 could be due to a high degree of non-neutralising antibodies, or cross-reactivity with other flaviviruses in the competitive ELISA, which in this context are considered as false positive reactions. Interestingly, this cross-reactivity seemed more pronounced in 2022, compared to other years. The results should also be viewed in the light of the complications for sero-surveillance of orthoflaviviruses, i.e. it is well-established that these viruses (e.g. WNV, USUV, tickborne encephalitis virus (TBEV)) can cross-react serologically in ELISA [35,41]. The risk of cross-reaction has been described as higher for IgG assays (which was used for bird samples in this study), when compared to IgM assays. Hence, interpretation of results obtained by ELISA serology should be considered with caution [41] and samples positive in ELISA should be confirmed by more specific tests, e.g. VNTs. Also, even for VNTs cross-reactions can occur between WNV and USUV, but specific antibodies can be attributed to one of the two viruses if the neutralising titre is fourfold or higher for one virus compared with the other when independently tested [35]. In Denmark TBEV is present [42], and USUV was just detected in 2024 within the framework of the Danish WNV surveillance system [43], potentially further complicating future serological surveillance for WNV. Optimally, the samples submitted from the migratory birds should be tested for antibodies against both these viruses, in addition to being tested in WNV VNT. However, the majority of the sampled bird species are very small in size (songbirds, weight between 10−30 grams), thus sufficient blood for follow-up testing is not always available. Also, even when tested in both WNV-VNT and USUV-VNT, some birds in our study had less than a four-fold difference in neutralisation titres between the two assays – thus a safe distinction between antibody responses to the two flaviviruses could not be made. A study from Germany reported that differentiation between WNV and USUV using VNTs is not always possible, and co-infections were proposed as a possible explanation (in one Eurasian blackbird and one house sparrow), since the neutralising titres were high against both viruses in these two birds [44]. In the current study, we did not observe high neutralising titres against both viruses in the same birds.

In addition to migratory birds, we also detected WNV-specific antibodies in one chicken, originating from the eastern part of Denmark. Birds that recover from infection with WNV usually develop a long-term immunity and it is believed that detection of antibodies against the virus in a resident bird population is indicative of local virus transmission [45]. Following experimental infection, it has been shown that chickens (obtained from specific pathogen-free hatching eggs from Germany) developed a low level of viraemia, but this level of infection may be insufficient to infect mosquitoes [46]. Sixty follow-up serum samples collected in 2020 from free-ranging poultry in the same area from Bornholm tested negative for antibodies using the competitive ELISA (data not shown). The following year, in 2021, it was attempted to investigate the presence of vector-competent mosquitoes in the same area (to collect evidence for the risk of local transmission), however, these samplings were unsuccessful due to the weather conditions in 2021. Given the limited possibilities for follow-up analysis in the flock, we cannot rule out that there was more than one infection on the island. Up until now, we have had no virus-positive findings from the wild bird population sampled on the island (Figure, panel C), but we have detected WNV specific antibodies in migratory birds sampled from the ringing site at a small island east of Bornholm. The role of migratory birds in the dispersal of WNV into new locations is known to be of high importance [47], thus, it is highly relevant to continue surveillance and testing of this bird category.

The results obtained under the current surveillance programme, i.e. the lack of detection of WNV and USUV in Denmark, should be viewed in the light of some of its limitations, especially, the relatively low number of samples tested each year within each sample category. For most samples (e.g. wild birds, poultry and bats), sample submissions depended on surveillance programmes established for other diseases (e.g. influenza in wild birds and poultry, rabies in bats, vector surveillance for mosquitoes). Therefore, samples numbers and sampling protocols were dependent on the outline of these programmes and were not planned *per se* for surveillance for WNV. For the vector population, trapping was primarily designed for assessing the vector population dynamics, and not for pathogen detection. Hence, collected *Cx. pipiens* and *Cx*. *torrentium* mosquitoes had potentially been exposed to degradation during trapping (e.g. traps were emptied on a weekly basis), which is not optimal for pathogen detection. *Culex modestus*, which were trapped by human landing catch, were not at the same risk of degradation. As part of the OH4Surveillance EU (HaDEA) project, a new risk-based targeted surveillance for WNV in mosquitoes has been initiated from the 2024 transmission season. The project targets areas of Denmark bordering Germany, and sampling protocols have been adapted to detect the pathogens in collected mosquitoes – e.g. emptying traps every day and keeping collected mosquitoes frozen according to the European Centre for Disease Prevention and Control (ECDC) technical report on field sampling of vectors [48].

Finally, a limitation of the programme is the lack of active surveillance in horses (a bona fide sentinel species) from the last 10 years of the programme. Given the proximity of symptomatic WNV cases in both birds and horses to the Danish−German border [17], initiating active surveillance in the horse population in Denmark would considerably increase surveillance sensitivity. In eastern-central Germany, non WNV-vaccinated horses have been described as useful sentinels for flavivirus circulation [18]. Besides re-instating active surveillance in the horse population, raised awareness of the disease among horse practitioners would hopefully result in more submissions from horses displaying neurological signs to the national reference laboratory for WNV analysis – submissions that have been very limited up until now.

## Conclusion

To our knowledge, this is the first study on WNV (and USUV) in the reservoir host and vector populations in Denmark. In the reporting period, from 2011−2023, we did not detect either WNV or USUV in Denmark, but the results support that incursion can occur. In the light of the current WNV and USUV circulation in regions close to the Danish southern borders, it is highly relevant to continue surveillance activities in the host- and vector populations in Denmark. Targeted activities to improve WNV surveillance in Denmark are currently being initiated. Despite the limitations of the currently implemented surveillance system, the detection of USUV in Denmark in 2024 within this system shows that it provides a useful framework to be built upon with new activities. The existing surveillance activities described here along with new targeted activities are of high importance to allow early warning and implementation of preventive measures in both human and animal populations.

## Data Availability

The data are available in the article and supplementary material.

## Disclaimer

Some of the data presented in the current publication has been published previously, in a preliminary form, in The Danish Veterinary Journal (in Danish) in 2012 (Polacek et al. “Antistoffer imod West Nile virus fundet i trækfugle i Danmark in 2011”), in 2016 (Lohse et al. ”West Nile Fever: En virussygdom, der spreder sig i Europa”) and in 2020 (Lohse & Bøtner ”Overvågning for West Nile Virus i fugle og myg i Danmark”).

## Supplementary Data

833000 SupplementaryMaterial
