# Supplementary material for: A decade of West Nile virus surveillance in the host and vector populations of Denmark, 2011 to 2023
Source: Euro Surveill. 2025 Sep 18;30(37):2400791. doi: 10.2807/1560-7917.ES.2025.30.37.2400791 (PMC12449679; doi:10.2807/1560-7917.ES.2025.30.37.2400791)
Supplement: SupplementaryMaterial [file 24-00791_SupplementaryMaterial.pdf]

This supplementary material is hosted by *Eurosurveillance* as supporting information alongside the article 'A decade of West Nile virus surveillance in the host and vector populations of Denmark, 2011 to 2023', on behalf of the authors, who remain responsible for the accuracy and appropriateness of the content. The same standards for ethics, copyright, attributions and permissions as for the article apply. Supplements are not edited by *Eurosurveillance* and the journal is not responsible for the maintenance of any links or email addresses provided therein.

Supplementary Table. Overview of 15 species of migratory birds in which antibodies against flaviviruses were detected using competitive ELISA, with findings stratified by year, Denmark, 2011–2023 (n = 3,053)

Year	*Curruca communis* common whitethroat			*Sylvia borin* garden warbler			*Phoenicurus phoenicurus* common redstart			*Phylloscopus trochilus* willow warbler			*Curruca curruca* lesser whitethroat		
	Number of samples: ELISA tested	Pos.	Prop. ELISA-pos. samples (95%CI)[a]	Number of samples: ELISA tested	Pos.	Prop. ELISA-pos. samples (95%CI)[a]	Number of samples: ELISA tested	Pos.	Prop. ELISA-pos. samples (95%CI)[a]	Number of samples: ELISA tested	Pos.	Prop. ELISA-pos. samples (95%CI)[a]	Number of samples: ELISA tested	Pos.	Prop. ELISA-pos. samples (95%CI)[a]
2011	14	0	0 (0-0.22)	9	1	0.11 (0.02-0.43)	19	1	0.05 (0.01-0.25)	0	0	NA	2	0	0 (0-0.66)
2012	8	1	0.12 (0.02-0.47)	31	1	0.03 (0.01-0.16)	23	0	0 (0- 0.14)	10	0	0 (0-0.28)	7	0	0 (0-0.35)
2013	25	5	0.20 (0.09-0.39)	34	2	0.06 (0.02-0.19)	65	0	0 (0- 0.06)	19	1	0.05 (0.01-0.25)	2	0	0 (0-0.66)
2014	16	0	0 (0-0.19)	44	2	0.05 (0.01-0.15)	57	0	0 (0-0.06)	79	0	0 (0-0.05)	5	0	0 (0-0.43)
2015	27	2	0.07 (0.02-0.23)	84	2	0.02 (0.01-0.08)	35	1	0.03 (0.01-0.15)	10	0	0 (0-0.28)	15	0	0 (0-0.20)
2016	10	1	0.10 (0.02-0.40)	30	3	0.10 (0.03-0.26)	88	1	0.01 (0-0.06)	89	0	0 (0-0.04)	14	0	0 (0-0.22)
2017	40	7	0.17 (0.09-0.32)	30	2	0.07 (0.02-0.21)	9	0	0 (0-0.30)	37	1	0.03 (0-0.14)	32	2	0.06 (0.02-0.20)
2018	20	2	0.10 (0.03-0.30)	52	1	0.02 (0-0.10)	98	2	0.02 (0.01-0.07)	60	0	0 (0-0.06)	23	1	0.04 (0.01-0.21)
2019	16	2	0.12 (0.03-0.36)	35	0	0 (0-0.10)	72	2	0.03 (0.01-0.10)	91	6	0.07 (0.03-0.14)	43	2	0.05 (0.01-0.15)
2020	6	0	0 (0-0.39)	33	1	0.03 (0.01-0.15)	83	2	0.02 (0.01-0.08)	68	2	0.03 (0.01-0.10)	25	0	0 (0-0.13)
2021	34	3	0.09 (0.03-0.23)	28	2	0.07 (0.02-0.23)	81	4	0.05 (0.02-0.12)	54	2	0.04 (0.01-0.13)	62	1	0.02 (0-0.09)
2022	22	7	0.32 (0.16-0.53)	11	3	0.27 (0.10-0.57)	67	3	0.04 (0.02-0.12)	34	2	0.06 (0.02-0.19)	15	6	0.40 (0.20-0.64)
2023	24	3	0.12 (0.04-0.31)	21	0	0 (0-0.15)	23	0	0 (0-0.14)	34	0	0 (0-0.10)	19	0	0 (0-0.17)
**Total:**	**262**	**33**	**0.13 (0.09-0.17)**	**442**	**20**	**0.05 (0.03-0.07)**	**720**	**16**	**0.02 (0.01-0.04)**	**585**	**14**	**0.02 (0.01-0.04)**	**264**	**12**	**0.05 (0.03-0.08)**

Year	*Lanius collurio* red-backed shrike			*Anthus trivialis* tree pipit			*Muscicapa striata* spotted flycatcher			*Sylvia atricapilla* blackcap			*Hippolais icterina* icterine warbler		
	Number of samples: ELISA tested	Pos.	Prop. ELISA-pos. samples (95%CI)[a]	Number of samples: ELISA tested	Pos.	Prop. ELISA-pos. samples (95%CI)[a]	Number of samples: ELISA tested	Pos.	Prop. ELISA-pos. samples (95%CI)[a]	Number of samples: ELISA tested	Pos.	Prop. ELISA-pos. samples (95%CI)[a]	Number of samples: ELISA tested	Pos.	Prop. ELISA-pos. samples (95%CI)[a]
2011	1	1	1[b] (0.21- 1)	1	0	0 (0-0.79)	0	0	NA	6	0	0 (0-0.39)	11	0	0 (0-0.26)
2012	5	0	0 (0-0.43)	1	0	0 (0-0.79)	35	1	0.03 (0.01-0.15)	25	1	0.04 (0.01-0.20)	20	0	0 (0-0.16)
2013	11	0	0 (0-0.26)	6	0	0 (0-0.39)	4	1	0.25 (0.05-0.70)	2	0	0 (0-0.66)	8	0	0 (0-0.32)
2014	8	1	0.12 (0.02-0.47)	14	0	0 (0-0.22)	19	0	0 (0-0.17)	5	0	0 (0-0.43)	2	0	0 (0-0.66)
2015	22	6	0.27 (0.13-0.48)	10	0	0 (0-0.28)	17	0	0 (0-0.18)	5	0	0 (0-0.43)	13	0	0 (0-0.23)
2016	3	0	0 (0-0.56)	4	0	0 (0-0.49)	8	0	0 (0-0.32)	0	0	NA	3	0	0 (0-0.56)
2017	5	1	0.20 (0.04-0.62)	3	0	0 (0-0.56)	5	0	0 (0-0.43)	37	1	0.03 (0-0.14)	22	0	0 (0-0.15)
2018	2	0	0 (0-0.66)	5	0	0 (0-0.43)	9	0	0 (0-0.30)	28	0	0 (0-0.12)	0	0	NA
2019	2	0	0 (0-0.66)	10	1	0.10 (0.02-0.40)	4	0	0 (0-0.49)	18	0	0 (0-0.18)	4	0	0 (0-0.49)
2020	5	1	0.20 (0.04-0.62)	5	1	0.20 (0.04-0.62)	13	0	0 (0-0.23)	0	0	NA	6	0	0 (0-0.39)
2021	2	0	0 (0-0.66)	11	2	0.18 (0.05-0.48)	6	0	0 (0-0.39)	15	0	0 (0-0.20)	8	0	0 (0-0.32)
2022	0	0	NA	7	1	0.14 (0.03-0.51)	10	2	0.20 (0.06-0.51)	30	1	0.03 (0.01-0.17)	16	3	0.19 (0.07-0.43)
2023	0	0	NA	5	0	0 (0-0.43)	7	0	0 (0-0.35)	45	1	0.02 (0-0.12)	17	0	0 (0-0.18)
**Total:**	**66**	**10**	**0.15 (0.08-0.26)**	**82**	**5**	**0.06 (0.03-0.13)**	**137**	**4**	**0.03 (0.01- 0.07)**	**216**	**4**	**0.02 (0.01-0.05)**	**130**	**3**	**0.02 (0.01-0.07)**

Year	*Oenanthe oenanthe* northern wheatear			*Phylloscopus sibilatrix* wood warbler			*Carpodacus erythrinus* common rosefinch			*Ficedula hypoleuca* European pied flycatcher			*Jynx torquilla* Eurasian wryneck		
	Number of samples: ELISA tested	Pos.	Prop. ELISA-pos. samples (95%CI)[a]	Number of samples: ELISA tested	Pos.	Prop. ELISA-pos. samples (95%CI)[a]	Number of samples: ELISA tested	Pos.	Prop. ELISA-pos. samples (95%CI)[a]	Number of samples: ELISA tested	Pos.	Prop. ELISA-pos. samples (95%CI)[a]	Number of samples: ELISA tested	Pos.	Prop. ELISA-pos. samples (95%CI)[a]
2011	0	0	NA	0	0	NA	0	0	NA	7	0	0 (0-0.35)	0	0	NA
2012	3	0	0 (0-0.56)	2	0	0 (0-0.66)	9	0	0 (0-0.30)	3	0	0 (0-0.56)	0	0	NA
2013	0	0	NA	0	0	NA	1	0	0 (0-0.79)	7	0	0 (0-0.35)	2	0	0 (0-0.66)
2014	0	0	NA	0	0	NA	0	0	NA	17	0	0 (0-0.18)	0	0	NA
2015	0	0	NA	1	0	0 (0-0.79)	0	0	NA	4	0	0 (0-0.49)	0	0	NA
2016	0	0	NA	7	1	0.14 (0.03-0.51)	0	0	NA	25	0	0 (0-0.13)	0	0	NA
2017	0	0	NA	1	0	0 (0-0.79)	0	0	NA	3	0	0 (0-0.56)	0	0	NA
2018	2	2	1 (0.34-1)	2	0	0 (0-0.66)	0	0	NA	6	0	0 (0-0.39)	0	0	NA
2019	0	0	NA	2	0	0 (0-0.66)	0	0	NA	2	0	0 (0-0.66)	1	0	0 (0-0.79)
2020	2	0	0 (0-0.66)	0	0	NA	0	0	NA	1	0	0 (0-0.79)	4	0	0 (0-0.49)
2021	1	0	0 (0-0.79)	1	0	0 (0-0.79)	0	0	NA	15	0	0 (0-0.20)	1	0	0 (0-0.79)
2022	0	0	NA	0	0	NA	1	1	1[b] (0.21-1)	8	1	0.12 (0.02-0.47)	3	1	0.33 (0.06-0.79)
2023	0	0	NA	1	1	1[b] (0.21-1)	0	0	NA	2	0	0 (0-0.66)	2	0	0 (0-0.66)
**Total:**	**8**	**2**	**0.25 (0.07-0.59)**	**17**	**2**	**0.12 (0.03-0.34)**	**11**	**1**	**0.09 (0.02-0.38)**	**100**	**1**	**0.01 (0-0.05)**	**13**	**1**	**0.08 (0.01-0.33)**

CI: Confidence interval, ELISA: enzyme-linked immunosorbent assay, N: number of samples; NA: not applicable; pos.: positive; prop.: proportion.

[a] Proportion of samples that tested positive in flavivirus ELISA, together with the 95 % confidence intervals (in parenthesis).

[b] Note that bird species in which a high proportion of birds tested positive corresponded to species with fewer samplings – as also indicated by wider 95 % confidence intervals, i.e. the high proportion of red-backed shrike (*Lanius collurio*), wood warbler (*Phylloscopus sibilatrix*), common rosefinch (*Carpodacus erythrinus*) testing positive in competitive ELISA in 2011, 2023 and 2022, respectively, is connected to sampling of only single individuals in those years (one positive of one tested).
